# Laparoscopic Roux-en-Y Gastric Bypass Without Division of the Mesentery Reduces the Risk of Postoperative Complications

**DOI:** 10.1007/s00464-018-6581-6

**Published:** 2018-11-20

**Authors:** Olof Backman, Jacob Freedman, Richard Marsk, Henrik Nilsson

**Affiliations:** 1 Department of Surgical and Perioperative Science (Hand and Plastic Surgery), Umeå University, Umeå, Sweden; 2 Division of Surgery, Department of Clinical Sciences, Danderyd Hospital, Karolinska Institutet, Stockholm, Sweden

**Keywords:** Gastric bypass, LRYGB, Leakage, Stenosis, Ulceration

## Abstract

**Background:**

Anastomotic complications after laparoscopic Roux-en-Y gastric bypass (LRYGB) including leaks, ulceration, and stenosis remain a significant cause of post-operative morbidity and mortality. Our objective was to compare two different surgical techniques regarding short-term anastomotic complications.

**Methods:**

A retrospective analysis of all patients operated with a primary LRYGB from 2006 to June 2015 in one institution, where prospectively collected data from an internal quality registry and medical journals were analyzed.

**Results:**

In total, 2420 patients were included in the analysis. 1016 were operated with a technique where the mesentery was divided during the creation of the Roux-limb (DM-LRYGB) and 1404 were operated with a method where the mesentery was left intact (IM-LRYGB). Leakage in the first 30 days [2.6% vs. 1.1% (*p* < 0.05)], and ulceration or stenosis occurring during the first 6 months after surgery [5.6% vs. 0.1% (*p* < 0.05)] was significantly higher in the DM-LRYGB group. Adjusted odds ratio for anastomotic leak was 0.46 (95% CI 0.24–0.87) and for stenosis/ulceration 0.01 (95% CI 0.002–0.09).

**Conclusion:**

IM-LRYGB seems to reduce the risk of complications at the anastomosis. A plausible explanation for this is that the blood supply to the anastomosis is compromised when the mesentery is divided.

Bariatric surgery has positive effects on overall survival and reduces obesity-related comorbidities such as myocardial infarction, stroke, and diabetes [1]. Since this type of surgery has rapidly increased in the last two decades, the surgical technique has developed. There is a wide spectrum of surgical procedures performed to achieve weight-loss, and each procedure has its own spectrum of complications and results. In Sweden the most popular procedure today is the laparoscopic Roux-en-Y gastric bypass (LRYGB) [2]. The most feared short-term complication after LRYGB is anastomotic leakage, which is reported to occur in 0.6–4.4% [3]. Later anastomotic complications are ulceration and stenosis in the gastro-enteroanastomosis (GE), where rates of ulceration vary between 0.6 and 25% [4] and strictures are reported in as much as 11.1% of patients in some studies [5].

LRYGB is commonly performed by the initial creation of a small gastric pouch, followed by the creation of a Roux-limb. This is traditionally accomplished by transection of the proximal jejunum about 30–50 cm distal to the ligament of Treitz, followed by division of the jejunal mesentery, typically using linear stapling devices. This technique is subsequently referred to as divided mesentery LRYGB (DM-LRYGB). A GE is constructed by approximation of the distal tip of the transected jejunum to the small gastric pouch, which is then anastomosed together, again typically using linear stapling devices. The Roux-en-Y reconstruction is completed by the creation of an entero-enteroanastomosis (EEA) between the proximal, or biliary, end of the transected jejunum and the Roux-limb, typically about 100 cm distal to the GE [6]. There are numerous variations in the basic LRYGB technique described above, for example, the Roux-limb can be brought to the gastric pouch in both an antecolic and retrocolic fashion, the GE can be constructed with a circular stapler, or even completely hand-sewn.

An alternative technique of creating the Roux-limb was presented in 2003 by a team from Gothenburg, here referred to as the Olbers/Lonroth, or intact mesentery LRYGB (IM-LRYGB), method [7]. The method creates an antecolic Roux-en-Y limb by first bringing a proximal jejunal loop up to the gastric pouch to create a side-to-side GE, without division of the mesentery, but originally described with mandatory division of the omentum to reduce traction at the site of the GE. Subsequently, the EEA is created and the Roux-en-Y construction is completed by the division of the jejunal loop between the GE and EEA, as described in Fig. 1. This IM-LRYGB technique was quickly adopted by almost all centers performing bariatric surgery in Sweden. At Danderyd Hospital, a DM-LRYGB technique was used until September of 2010 when it was changed to the IM-LRYGB technique, since centers less experienced in bariatric surgery using the IM-LRYGB technique showed lower leakage rates in the Scandinavian Obesity Surgery Registry (SOReg). Both anastomotic leaks and stenosis or ulcerations are complications that at least partially can be regarded as ischemia-related.

**Fig. 1:**
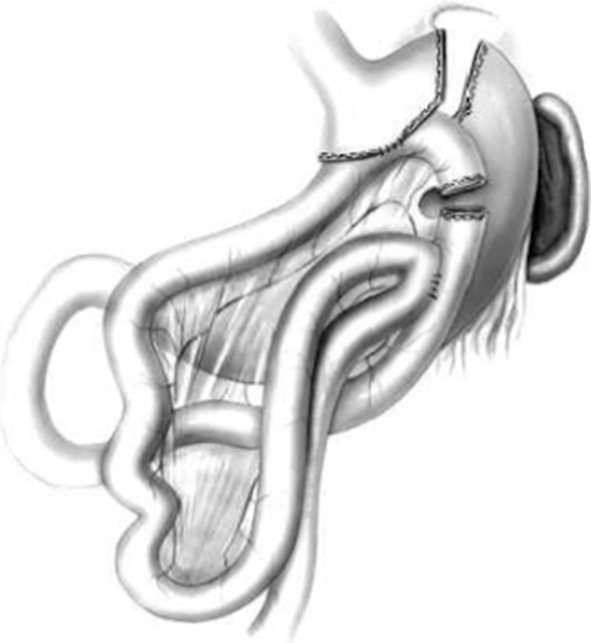
The principle behind the intact mesentery LRYGB (IM-LRYGB) (Figure from original article describing the method [7])

The aim of this study was to compare the outcome of two different techniques for LRYGB used in a single high-volume surgical center, with specific emphasis on anastomotic leaks and clinically significant strictures or ulcerations in the GE.

## Materials and methods

Prospectively collected data from an internal quality registry of all bariatric procedures performed at Danderyd Hospital and patients’ medical records were retrospectively reviewed. Data on pre-operative BMI, age, gender, post-operative complications, surgical method, reoperations, and endoscopies were collected and analyzed. All operations were performed according to standardized methods. The study was approved by the Regional ethical review board in Stockholm.

### Patients

All patients treated with a primary antecolic LRYGB at Danderyd Hospital from 2006 to June 30th 2015 were eligible for inclusion in the study. All patients in the study were followed for 6 months.

### Surgical procedures

For both procedures, a biliary limb of approximately 30–50 cm was created and the Roux-limb was measured to be approximately 100 cm. Regardless of procedure (IM-LRYGB or DM-LRYGB), both anastomoses were created with a single 45 mm linear stapler with closure of the enterotomy with a running Vicryl 3-0 suture. The IM-LRYGB method used was slightly modified compared to the originally described Olbers/Lonroth method [7]. For example, the division of the omentum was only performed when deemed necessary according to the judgment of the operating surgeon. Also the proximal biliary or jejunal limb was regularly divided immediately after creation of the GE and before the creation of the EEA, and not as a final step after creation of both anastomoses as in the originally described method. At the end of the operation, a leakage test by insufflation of air into the gastric pouch, immersed under a saline solution, was performed in all cases.

### Pre- and post-operative care

Patients in both groups routinely received pre-operative antibiotic prophylaxis, antithrombotic prophylaxis with low molecular-weight heparin (LMWH) for 10 days, and post-operative proton pump inhibitors (PPI). Initially, patients were treated with PPI for 1 month post-operatively, but the routine was adjusted to a 3 month post-operative PPI treatment regime from October 2010.The presence of Helicobacter Pylori (HP) was not routinely tested for, so HP status was unknown in both groups. Patients were strictly advised to quit smoking at least 4 weeks pre- and post-operatively, but no formal testing for compliance to this advice was performed.

### Definition of outcome

The outcomes studied were leakage in either the GE or EEA during the first 30 post-operative days, and stenosis or ulceration occurring in the GE during the first 6 months after surgery. Anastomotic leakage was defined as any sign of a leak requiring intervention with endoscopy, drainage, or reoperation where a leak was either confirmed at reoperation or by radiologic studies. Stenosis and ulceration was defined as stenosis or ulceration verified with endoscopy, and the events were analyzed as one entity. Since only patients with symptoms indicating GE-problems were examined with endoscopy, only symptomatic ulcers or GE strictures were regarded as events.

### Statistical analysis

Descriptive statistics were used for presentation of patient characteristics and numbers are presented either as means with standard deviation or as proportions. Differences between groups were analyzed using the two-sample *t*-test or Pearson Chi^2^ test as appropriate. Failure-curves were calculated using Kaplan–Meier estimates. Independent predictors of complications were identified and odds ratios (OR) calculated using multivariate logistic regression. The threshold for statistical significance was set to *α* < 0.05. STATA 14 (StataCorp, College Station, Texas 77845 USA) was used for the statistical analyses.

## Results

In total, 2949 patients underwent a primary antecolic LRYGB at Danderyd Hospital during the study period. To minimize the effect of learning-curve bias 329 patients operated by surgeons with less than 100 LRYGB procedures during this period were excluded from the analysis. For the same reason, the first 100 patients operated with each technique were also excluded prior to analysis, leaving 2420 patients included in the data analysis. 1016 of the included patients had surgery between December 2006 and September 2010 with DM-LRYGB and 1404 patients had surgery between November 2010 and June 2015 with the IM-LRYGB method.

Clinical characteristics of the patients are presented in Table 1. The patients in the DM-LRYGB were slightly older and had a somewhat higher BMI pre-operatively, however, the differences were small. The operating time was longer in the DM-LRYGB group, and even if the conversion rate to open surgery was low in both groups, it was slightly lower in the IM-LRYGB group.

**Table 1:** Patient characteristics

	DM-LRYGB (*n* = 1016)	IM-LRYGB (*n* = 1404)	*p*
Women (%)	74.3	74.6	0.88
Age (years); (mean (SD))	43.0 (11.4)	41.7 (11.6)	0.05
BMI (kg/m^2^); (mean (SD))	42.1 (6.0)	39.4 (5.3)	0.05
Operation time (min); (mean (SD))	71.6 (25.2)	58.2 (20.0)	0.05
Completed laparoscopic surgery (%)	98.2	99.3	0.05

*DM-LRYGB* divided mesentery laparoscopic Roux-en-Y gastric bypass, *IM-LRYGB* intact mesentery laparoscopic Roux-en-Y gastric bypass, *BMI* Body Mass Index, *SD* standard deviation

**Fig. 2:**
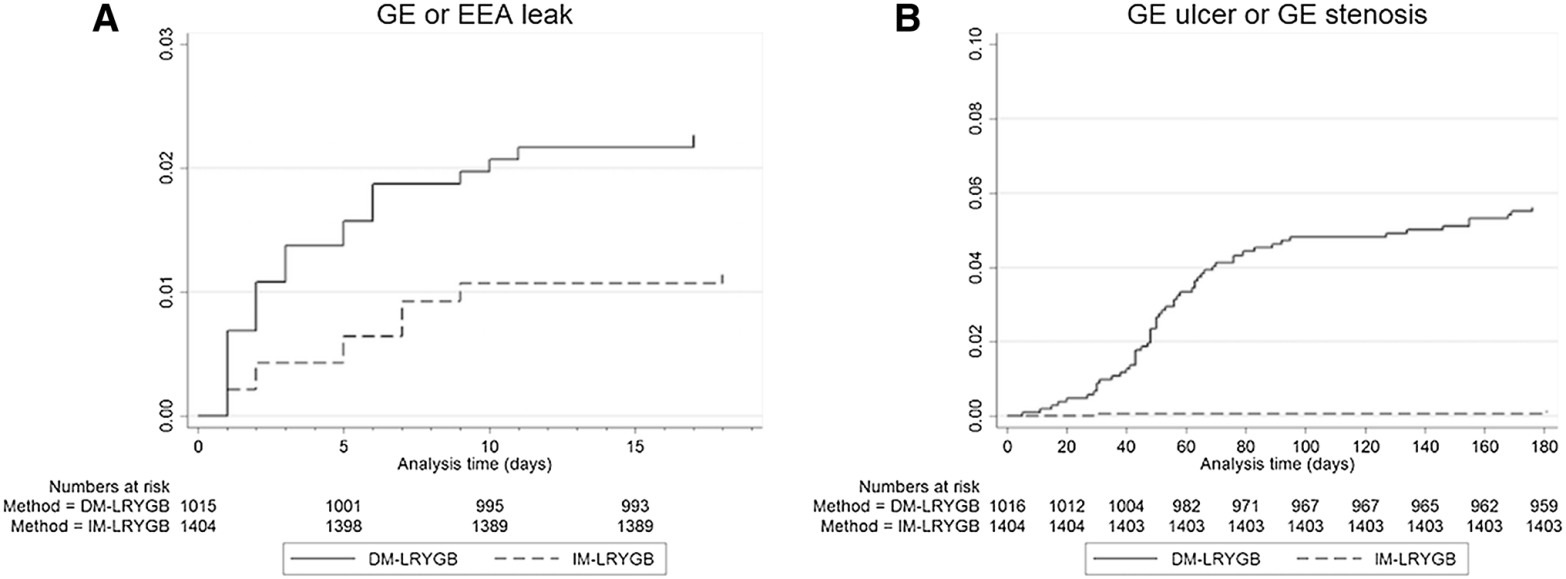
Kaplan–Meier failure-curves showing the rates of anastomotic leaks in (**A**) and clinical stenosis or ulcerations in (**B**). *IM-LRYGB* intact mesentery laparoscopic Roux-en-Y gastric bypass, *DM-LRYGB* divided mesentery laparoscopic Roux-en-Y gastric bypass

The rate of GE leakage was reduced after the change of surgical technique, from 2.6% in the DM-LRYGB group to 1.1% in the IM-LRYGB group (*p* < 0.05). The rate of stenosis or ulceration was significantly improved, from 5.6% in the DM-LRYGB group to 0.1% in the IM-LRYGB group (*p* < 0.05). In multivariate regression analysis, the adjusted odds ratio for leakage in the IM-LRYGB group was 0.46 (95% CI 0.24–0.87), and for stenosis or ulceration the odds ratio was 0.01 (95% CI 0.002–0.09). Detailed results from the multivariate regression are presented in Table 2. As shown in Fig. 2, the majority of the leaks were diagnosed during the first post-operative week, and the majority of GE strictures or ulcerations were diagnosed during the first 3 months.

**Table 2:** Multivariate regression analysis

	Leakage	Stenosis/ulceration
	OR (95% CI)	OR (95% CI)
Surgical technique (IM-LRYGB vs. DM-LRYGB)	0.46 (0.24–0.87)	0.01 (0.002–0.09)
Age	1.05 (1.02–1.08)	1.05 (1.02–1.07)
Gender (male vs. female)	1.50 (0.79–2.86)	1.04 (0.57–1.89)
BMI	0.98 (0.93–1.04)	0.99 (0.94–1.04)

*IM-LRYGB* intact mesentery laparoscopic Roux-en-Y gastric bypass, *DM-LRYGB* divided mesentery laparoscopic Roux-en-Y gastric bypass, *BMI* Body Mass Index

## Discussion

This study indicates that anastomotic complications, such as leakage, ulceration, and stenosis, might be reduced by not dividing the mesentery when performing LRYGB, at least in combination with a prolonged PPI treatment. By avoiding division of the mesentery, the risk for ischemic complications in the anastomosis may be reduced. It is well known that ischemia affect healing and increases the risk for leakage in gastrointestinal anastomoses [8, 9]. The rate of leakage after LRYGB varies in the literature [3], recently the multicenter LABS study presented low leakage rates of 0.8% in primary LRYGB [10] and similar leakage rates was reported in the nationwide Swedish SOReg report from 2015 (0.96% for patients operated between 2012 and 2014) [2]. This is about the same leakage rate as found in this study with the IM-LRYGB technique. There are many factors that can affect leakage rates. For example, adverse events have been shown to decrease with increased surgical volume and there seems to be a tendency for lower leakage rates in later studies [11]. Some also present lower leakage rates toward the end of published series [12] and all this could indicate that there is a significant learning-curve, which could influence the results of the present study as the patients in the IM-LRYGB group underwent surgery in a more recent time.

GE stenosis is not as dramatic as a GE leakage and is most often treatable with repeated endoscopic interventions [13, 14], but treatment with dilatation can cause perforation in rare cases [5, 13]. There are several factors that can affect the rate of ulceration and stenosis in the anastomosis, for example smoking [4], size of the gastric pouch [4], previous ulcer disease [15], PPI treatment [16], diabetes [15], and type of stapler device used [17–19]. A possible explanation for stenosis is that it is actually a late complication to an early ulceration, which subsequently heals with stenosis. This theory is supported by the fact that 40% of patients with GE ulcer on routine endoscopy after LRYGB had a partial stenosis at the same time [20]. Stricture and ulceration rates with the DM-LRYGB technique in the present study are similar to stricture rates presented in many other studies [13, 14, 17, 21].

The rate of ulceration in the literature varies from 0.6 to 25%, with many studies reporting numbers in par with the results in the DM-LRYGB group in the present study [4]. In a study where endoscopy was routinely performed after gastric bypass, ulcerations were found in 12.3% of LRYGB patients, and it was also noted that 28% of patients with marginal ulcer were asymptomatic [20]. Even in severe cases with perforating ulcers, 20% of the patients had no previous symptoms [22]. Mucosal ischemia occurs after gastrointestinal stapling and creation of anastomoses [9]. The theory that ischemia is an important factor for ulcers and stenosis is somewhat supported by the fact that ulceration is more common in diabetic patients [15, 23] and that ulceration and stenosis seem to be an early complication, maybe before angiogenesis occur. Intuitively, the circulation to the anastomosis could be compromised when the mesentery is divided and sometimes it is visible during DM-LRYGB surgery that the intestine looks a bit strained when the mesentery is divided and the intestine is pulled up to reach the gastric pouch.

Data on certain known risk factors for anastomotic complications such as diabetes, smoking, the use of non-steroid anti-inflammatory (NSAID) drugs, and the size of the gastric pouch were not available in the present study, and could therefore not be controlled for. It could be assumed with reasonable certainty though, that these possible confounders would be equally distributed between the two historic cohorts. The cohorts originate from the same area, the indication for surgery did not change during the study period, nor did the surgical technique regarding construction of gastric pouches change. According to the SOReg the overall rate of pre-operative diabetes was the same in Sweden 2010 as in 2016 indicating that the group of patients has not changed much over time.

As shown in Table 2, the conversion rate was 1.8% during the first period and 0.7% during the second period. This is almost exactly the same conversion rates as seen in reports from SOReg, where a decreasing conversion rate can be observed over time. Reasons for conversion could be adhesions from previously performed abdominal surgery (not bariatric though), tight mesentery, bleeding complications, and other miscellaneous reasons.

Apparent weaknesses of this study is the retrospective design with comparison of two historic cohorts, and the risk that learning-curve bias cannot be excluded, even though an attempt to reduce this risk was done by the exclusion of the first 100 patients in each group. The team of surgeons involved also had long experience with both open and laparoscopic bariatric surgery at the start of the study and they had performed LRYGB surgery for several years before the study started.

Since this is a retrospective study based on a database and electronic patient records, one can argue that there was a 100% follow-up rate at both 30 days and 6 months since no patients were lost to follow-up. On the other hand, since patients were not actively contacted as would have been the case in a prospective study, it is not known if patients were admitted to hospitals outside of the Stockholm County Council area, which is the catchment area covered by the electronic patient records, and treated for complications elsewhere. One can therefore also argue that we the actual follow-up rates are unknown. Although uncertain, there is no substantial reason to believe that the proportion of complications possibly missed would differ between the two time-periods, thereby introducing significant bias that could influence the results in this study.

Another concern is that at the same time as operating technique was changed from DM-LRYGB to IM-LRYGB, the post-operative PPI treatment was extended from 1 to 3 months, which may have influenced the rates of ulceration and stenosis in this material, since a recent retrospective study shows that ulceration at the anastomosis is reduced after extension of PPI treatment from 1 to 3 months [16]. The difference in leakage rates observed could not have been affected by this change in practice, since the PPI treatment was the same in both groups during the first 30 days. The stenosis and ulceration rate during ongoing PPI treatment are also lower in the IM-LRYGB group and this indicate that prolonged PPI is not the only explanation for the significant reduction in stenosis and ulceration. Earlier studies on the effect of prolonged duration of PPI treatment also show a more moderate effect on ulceration compared to the results from the present study [16]. This could indicate that the IM-LRYGB technique and prolonged PPI are two separate factors that reduce the risk for anastomotic complications.

Another complication after LRYGB that is not studied in the present study is internal herniation (IH). The literature regarding IH after IM-LRYGB is a bit conflicting. A retrospective study of 1400 patients present IH in only 0.2% when performing the IM-LRYGB even if the mesenteric defects are left open, possibly by reducing the size of the Peterson defect [24]. However, the follow-up time in this study is short and other case series show higher rates of IH at a later time point [25]. Another study on this matter retrospectively compared DM-LRYGB with IM-LRYGB, showing a dramatic decrease in IH in favor for the IM-LRYGB. The results in this study must also be interpreted with caution since the mesenteric defects was closed in the IM-LRYGB and not in the DM-LRYGB [26], and a recent randomized study show that closure of the mesenteric defects after IM-LRYGB reduces IH by about 50% [27].

The underlying idea that IM-LRYGB reduces the risk of ischemia-related complications in the GE is plausible. Even though the present study has some obvious shortcomings, the findings are quite dramatic, especially regarding ulceration and stenosis. The findings cannot easily be disregarded as being only the result of a learning-curve and prolongation of post-operative PPI treatment. Our conclusion from this study is that LRYGB should be performed without division of the mesentery to reduce the risk of leakage in the anastomosis, and in combination with a prolonged PPI treatment it also reduces the risk of stenosis and ulceration in the gastro-enteroanastomosis. The IM-LRYGB technique and the prolonged PPI treatment probably have separate or additive risk-reducing effects on ulceration and stenosis.
